# Squamous Cell Carcinoma Originating from Adult Laryngeal Papillomatosis: Case Report and Review of the Literature

**DOI:** 10.1155/2018/4362162

**Published:** 2018-12-19

**Authors:** Vivian Narana Ribeiro El-Achkar, Andressa Duarte, Fabiano Pinto Saggioro, Francisco Veríssimo De Mello Filho, Jorge Esquiche León, Alfredo Ribeiro-Silva, Estela Kaminagakura

**Affiliations:** ^1^DDS, PhD Student at Department of Bioscience and Oral Diagnosis, Science and Technology Institute, Universidade Estadual Paulista (UNESP), Sao Jose dos Campos, Brazil; ^2^PhD Student at Department of Pathology and Legal Medicine, Ribeirão Preto Medical School, University of São Paulo, Ribeirao Preto, Brazil; ^3^PhD, Assistant Physician at Pathology Service of Department of Pathology and Legal Medicine, Ribeirão Preto Medical School, University of São Paulo, Ribeirao Preto, Brazil; ^4^MD, PhD, Associated Professor at Department of Ophthalmology, Otorhinolaryngology and Head and Neck Surgery, Ribeirão Preto Medical School, University of São Paulo, Ribeirão Preto, Brazil; ^5^PhD, Professor at Department of Stomatology, Collective Health and Legal Dentistry, Ribeirão Preto Medical School, University of São Paulo, Ribeirao Preto, Brazil; ^6^MD, PhD, Associated Professor at Department of Pathology and Legal Medicine, Ribeirão Preto Medical School, University of São Paulo, Ribeirao Preto, Brazil; ^7^DDs, PhD, Associated Professor at the Department of Bioscience and Oral Diagnosis, Science and Technology Institute, Universidade Estadual Paulista (UNESP), Sao José dos Campos, Brazil

## Abstract

**Background:**

The malignant transformation of laryngeal papillomatosis (LP) into squamous cell carcinoma (SCC) can occur in up to 4% of LP cases. The low-risk HPV types 6 and 11 are those that are most commonly related to LP; however, high-risk HPV types may be present. The present study reviews the literature on cases of malignant transformation of LP in adults and reports a clinical case.

**Case Report:**

A 47-year-old male patient exhibiting hoarseness for 4 months presented an exophytic lesion in the right palatine tonsil and a digitiform-like lesion in the right vocal fold. The biopsy revealed a well-differentiated SCC in the vocal cord, which showed a transition zone with a squamous papillomatous lesion. By using the chromogenic *in situ* hybridization (CISH) test, both lesions showed a positive result for high-risk HPV types 16 and 18 and negative for low-risk HPV types 6 and 11. The final diagnosis was SCC arising from LP. The patient underwent surgical treatment. After 36 months of follow-up, no signs of recurrence were observed.

**Results:**

The literature review revealed 25 cases of malignant transformation into SCC of LP with adult onset. Of these, only 9 cases were assessed by CISH and/or PCR for HPV identification, of which 7 were positive. The current study focuses on the eighth case, suggesting the involvement of the high-risk HPV types in its pathogenesis.

**Conclusions:**

LP is considered a benign lesion with the potential for malignant transformation, which reinforces the need for its early diagnosis and the constant monitoring of patients with LP.

## 1. Introduction

Papilloma is the most common benign tumor affecting the larynx, which may present as a single event or as recurring and/or affecting more than one topography, as occurs in laryngeal papillomatosis (LP) [1]. LP is distributed bimodally, affecting juvenile patients (JLP), with onset of disease before 5 years of age and adult patients, aged 20–40 years (ALP) [1]. ALP is prevalent in male patients [1] and tends to be less aggressive than JLP [2].

ALP is considered to be the most important clinical manifestation of human papillomavirus (HPV) larynx infection and is correlated mainly with low-risk HPV's of malignancy types 6 and 11 [3]. The disease may be progressive, with a high relapse rate and requiring more than 100 surgical procedures, but spontaneous remission may also occur [4, 5].

Malignant transformation of LP is rare, having been described in about 1–4% of cases [1]. The following is a case of malignant transformation of LP in an adult patient with no history of recurrence, a rare case that shows that even nonrecurrent lesions have a potential for malignancy. We also performed a brief review of the literature, looking for cases of malignancy in patients who had suffered their first manifestation of the disease in adulthood.

## 2. Materials and Methods

A search for English language articles was carried out in the PubMed, Scopus, and Web of Science databases using the following keywords: malignancies in ALP and epidermoid carcinoma/squamous cell carcinoma from ALP. The search period extended from 1988 to the present, and only the reports in which the onset of the disease occurred in the adult phase were considered; cases in which malignancy occurred in patients of juvenile onset LP were discarded.

## 3. Case Report

**Figure 1:**
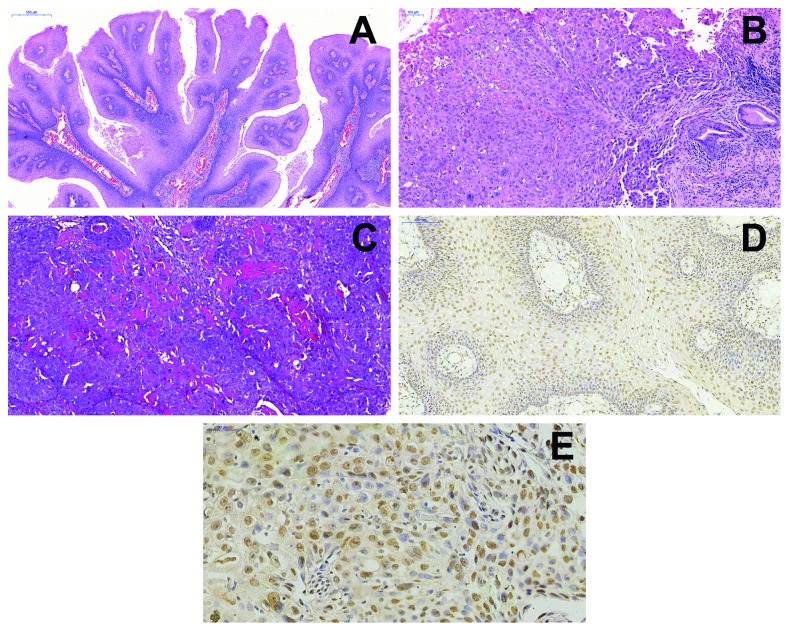
Photomicrographs: lesion with papillary appearance showing exophytic projections of epithelial proliferation with benign appearance (a), transition zone of malignant neoplasm in the right vocal fold (b), well-differentiated SCC (c), dotted brown nuclei showing positive CISH result for high-risk HPV (16/18) in LP (d), and in SCC (e).

A 47-year-old male patient noticed a change in his voice (hoarseness) 4 months previously. During the anamnesis, he did not report any addictions or habits, but systemic hypertension had been diagnosed and controlled. During intraoral physical examination, a pediculated exophytic lesion with a rough surface and coloration similar to adjacent mucosa was observed in the right tonsil. Examination by laryngoscopy revealed an exophytic lesion in the right paralyzed vocal fold, occupying its anterior two-thirds, in which the mucosa was covered by fibrinopurulent exudate. An incisional biopsy was performed on the vocal cord and excisional on the palatine tonsil. Microscopically, the vocal cord biopsy revealed a squamous epithelium exhibiting architectural disorganization, covered by a fibrinopurulent membrane. This epithelium exhibited projections towards the connective tissue and was infiltrated into the connective tissue, forming islands and strands of malignant epithelial cells (Figure 1).

The squamous layer showed clear epithelial cells with a vacuolized nuclei, and some cells similar to koilocytes were present in the upper layers of the epithelium. The epithelium was found with digitiform projections and fibrovascular connective tissue centers containing mononuclear inflammatory infiltrate (Figure 1(a)). A transitional zone was found in this biopsy, where the atypical epithelium protrudes exophytically with a digitiform aspect (Figure 1(b)). The malignant component showed atypical cells, with nuclear pleomorphism, sometimes binucleated cells, with a hyperchromatic nucleus and individual keratinization. Atypical mitoses and areas of necrosis were also found (Figure 1(c)). The result of the incisional biopsy was of a well-differentiated squamous cell carcinoma (SCC) present in the right vocal cord. Histological examination suggested that the SCC originated from a papillomatous lesion, and chromogenic in situ hybridization (CISH) was performed. Both lesions showed a positive result in CISH for high-risk HPV types 16 and 18 (Figures 1(d) and 1(e)) and negative for low-risk HPV types 6 and 11.

On the other hand, the histological sections of the right palatine tonsil region revealed a squamous papilloma demonstrated by proliferation of the stratified squamous epithelium, predominantly nonkeratinized, presenting exocytosis and basal layer hyperplasia with typical mitoses.

### 3.1. Chromogenic *in situ* Hybridization (CISH)

The following procedures were carried out from the biopsy block: dewaxing, peroxidase blockade, and enzymatic digestion, followed by a pretreatment with a 95% EDTA bath for 15 min, followed by several rinses with distilled water and dehydration. After drying, the probe was added onto the material, the slices were covered by a coverslip, and sealing was performed. The material was denatured for 5 min at 75°C, and hybridization was performed at 37°C for 60 min for high-risk HPV. The ZytoFast Plus CISH Implementation Kit-HRP-DAB (ZytoVision, Bremerhaven, Germany, and the ZytoFast HPV-type 6/11 Probe and 16/18 Probe) was used. In the next step, the coverslip was removed, and the slides were washed in TBS buffer at 55°C for 5 min, and then, the primary antibody was incubated at 37°C for 30 min in a humid chamber. For tagging, chromogen diaminobenzidine (DakoCytomation, Carpinteria, CA, USA) was used for 10 min, and Mayer's Hematoxylin was used for the counterstaining. Reactions were performed with negative and positive controls.

### 3.2. Treatment and Prognosis

Surgery was performed, including tracheostomy, with the final diagnosis of SCC originating from LP, staging T3N0M0. One month after the surgical procedure, the patient returned without complaints. In the other nasofibroscopy and bronchoscopy examinations, no changes were observed. Follow-up by a speech therapist was fundamental for significant voice improvement. The patient has been undergoing maintenance for 36 months and has no complaints and no signs of relapse.

### 3.3. Literature Review

Fifteen articles were found, totaling 27 cases of malignant transformation of LP with onset in adult patients. The mean age of the patients was 57.03 years (SD = 13.76). Of these, including the present case, 25 (89.28%) were male patients (Table 1), and in 3 (10.71%) cases, there was no history of recurrent LP. The localization of the carcinoma was cited in 10 cases, of which 3 (30%) were malignant lesions in the lung, due to dissemination. In the remaining 7 (70%), the larynx was affected, mainly in the vocal cords. There were 9 (47.36%) smokers, and HPV was identified in 9 of the reports, 2 negative tests, 4 with low-risk HPV and 4 with high-risk HPV.

## 4. Discussion

In adults, the exact mode of infection is controversial; transmission during sexual contact and reactivation of a slow-progressing latent infection acquired in childhood has been suggested [1]. The aggressive clinical course of LP can lead to malignant transformation and death by extending through the lower respiratory tract [1]. The rates of LP malignancy are around 1 to 4% [1]; however, there are few studies with an extensive review of these cases [21]. In this present review, only cases in which the disease started in adulthood were considered.

Gastroesophageal reflux and immunosuppression may also be aggravating factors for patients with papillomatosis [15, 16]. In the present case, no signs of reflux or immunosuppression were found. Tobacco use, irradiation [21], and p53 mutation [22] may also favor malignant transformation. However, it may also occur in the absence of these factors [23].

Pou et al. [11] reported that, of 17 patients who underwent a LP aggressive clinical course, the malignant transformation occurred in 3 of them, there being 2 of juvenile onset, with more than 100 relapses, and HPVs 6 and 11 were found. Another case occurred in the adult patient after 12 recurrences, and HPV 16 was identified. Our report highlights the rapid progression to SCC; the patient presented symptoms for only 4 months and the malignant lesion was diagnosed together with papillomatosis; it being able to attribute this to an aggressive progression of high-risk HPV 16. The rarity of cases in which the malignant transformation has a rapid course was observed; in 3 reported cases, the patients did not have recurrent disease, and in 1 case, the carcinoma was detected only 6 months after the diagnosis of LP [20]. Other factors associated with more aggressive LP are the higher number of recurrences and evolution time greater than 10 years [5].

The incidence of high-grade dysplasia for LP is between 10% and 17.6% [24–26]. The progression of LP with dysplasia with multiple recurrences to carcinoma was reported [10]. Hall et al. [27] reported that only one (2%) of the 54 adult patients with recurrent respiratory papillomatosis developed SCC. However, the patient had a history of smoking and had a severe dysplasia. In another study, there were 9 male patients with adult onset LP, which had transformed into SCC. Initially, in 2 cases, there was no dysplasia; 2 cases were diagnosed with a mild condition, 3 were moderate, and 2 exhibited severe dysplasia [19]. In the presented case, there was a papillomatosis lesion with low-grade dysplasia associated with carcinoma. Recently, squamous hyperplasia and mild dysplasia were grouped into low-grade lesions and moderate and severe dysplasia in high-grade lesions, according to the malignancy potential [1].

Pulmonary imaging (RX or CT or PET scan) is recommended, in addition to careful histopathological examination, which should be repeated every time a surgery is performed to remove papillomatosis. There should be routine following-up of patients with LP, since there are reports of pulmonary dissemination, which develops into malignancy in a short period of time and which, if detected early, guarantees a greater survival time for the patient [17]. Within 36 months of follow-up of the patient in the present study, there was no involvement of the lungs.

Although LP is a benign lesion, it can present a severe course with malignant transformation, especially when high-risk HPV is detected. Therefore, the follow-up of these patients is highly recommended.

## Figures and Tables

**Table 1:** Clinical information of adult patient's onset who developed SCC and was reported in the English language literature.

Author(s), year	Age	Gender	RRP	Risk factors	HPV
Byhardt et al, 1988 [6]	61	Male	Yes	Smoker and Rxt	NR
Kashima et al, 1988 [7]	28	Male	Yes	Rxt	6
Zarod et al, 1988 [8]	72	Female	Yes	NR	6
Lindeberg et al, 1989 [9]	62.5	Male	Yes	Smoker	Negative
Lindeberg et al, 1989 [9]	34.4	Male	Yes	NR	Negative
Hasan et al, 1995 [10]	30	Male	Yes	Nonsmoker, without Rxt	NR
Pou et al, 1995 [11]	46	Male	Yes	Nonsmoker	16
Sakakura et al, 1996 [12]	63	Female	Yes	Nonsmoker	18
Klozar et al, 1997 [13]	81	Male	Yes	NR	NR
Klozar et al, 1997 [13]	58	Female	Yes	NR	NR
Klozar et al, 1997 [13]	56	Male	No	NR	NR
Behl et al, 2001 [14]	28	Male	Yes	Nonsmoker	NR
Azadamaki et al, 2013 [15]	77	Male	Yes	Nonsmoker	NR
Hao et al, 2013 [16]	52	Male	No	Passive smoker	16/18
Hasegawa et al, 2013 [17]	75	Male	Yes	NR	11
Jeong et al, 2009 [18]	55	Male	Yes	NR	6, 11
Jeong et al, 2009 [18]	64	Male	Yes	NR	NR
Ilmarinen et al, 2014 [19]^*∗*^	—	Male	Yes	7 smokers, 2 nonsmokers	NR
Zhang et al, 2015 [20]	43	Male	No	Multiple sex partners, second-hand smoker	NR
Present study	47	Male	No	No	16/18

RRP: recurrent respiratory papillomatosis; Rxt: radiotherapy; NR: not reported. ^*∗*^The authors described 9 cases of malignancy, all were male and the mean age was 62 years (range: 41–79).
